# Chilean *Pitavia* more closely related to Oceania and Old World Rutaceae than to Neotropical groups: evidence from two cpDNA non-coding regions, with a new subfamilial classification of the family

**DOI:** 10.3897/phytokeys.19.3912

**Published:** 2012-12-18

**Authors:** Milton Groppo, Jacquelyn A. Kallunki, José Rubens Pirani, Alexandre Antonelli

**Affiliations:** 1 Departamento de Biologia, FFCLRP, Universidade de São Paulo, Av. Bandeirantes 3900, 14040-901 – Ribeirão Preto, SP, Brazil; 2 The New York Botanical Garden, Bronx, NY, 10458-5126, USA; 3 Instituto de Biociências, Universidade de São Paulo, Rua do Matão 277, 05508-090, São Paulo, SP, Brazil; 4 Department of Biological and Environmental Sciences, University of Gothenburg, Carl Skottsbergs gata 22B, PO Box 461, 405 30 Gothenburg, Sweden

**Keywords:** Biogeography, Cneoroideae, phylogeny, Pitavia, Rutaceae, Rutoideae, *rps16*, subfamily, *trnL-trnF*

## Abstract

The position of the plant genus *Pitavia* within an infrafamilial phylogeny of Rutaceae (rue, or orange family) was investigated with the use of two non-coding regions from cpDNA, the *trnL-trnF* region and the *rps16* intron. The only species of the genus, *Pitavia punctata* Molina, is restricted to the temperate forests of the Coastal Cordillera of Central-Southern Chile and threatened by loss of habitat. The genus traditionally has been treated as part of tribe Zanthoxyleae (subfamily Rutoideae) where it constitutes the monogeneric tribe Pitaviinae. This tribe and genus are characterized by fruits of 1 to 4 fleshy drupelets, unlike the dehiscent fruits typical of the subfamily. Fifty-five taxa of Rutaceae, representing 53 genera (nearly one-third of those in the family) and all subfamilies, tribes, and almost all subtribes of the family were included. Parsimony and Bayesian inference were used to infer the phylogeny; six taxa of Meliaceae, Sapindaceae, and Simaroubaceae, all members of Sapindales, were also used as out-groups. Results from both analyses were congruent and showed *Pitavia* as sister to *Flindersia* and *Lunasia*, both genera with species scattered through Australia, Philippines, Moluccas, New Guinea and the Malayan region, and phylogenetically far from other Neotropical Rutaceae, such as the Galipeinae (Galipeeae, Rutoideae) and Pteleinae (Toddalieae, former Toddalioideae). Additionally, a new circumscription of the subfamilies of Rutaceae is presented and discussed. Only two subfamilies (both monophyletic) are recognized: Cneoroideae (including Dictyolomatoideae, Spathelioideae, Cneoraceae, and Ptaeroxylaceae) and Rutoideae (including not only traditional Rutoideae but also Aurantioideae, Flindersioideae, and Toddalioideae). As a consequence, Aurantioideae (*Citrus* and allies) is reduced to tribal rank as Aurantieae.

## Introduction

Rutaceae is a large, predominantly tropical and subtropical family, consisting of 150–162 genera and 1500–2096 species, with three main centers of diversity: Tropical America, southern Africa, and Australia (Groppo 2010, Simpson 2010, Kubitzki et al. 2011). The family has long been economically important for edible fruits (especially *Citrus*, with many varieties of oranges, lemons, tangerines, etc.), aromatic oils (*Boronia* and *Ruta*), drugs (e.g., *Pilocarpus*, source of pilocarpine, used against glaucoma), and bitter beverages used to treat fevers (*Angostura*, *Galipea*). Species of *Flindersia*, *Zanthoxylum*, *Balfourodendron*, and *Euxylophora* are sources of timbers. More recently, the antimicrobial and antifungal properties of rutaceous compounds are being exploited as natural pesticides (e.g., Oliva et al. 2000), herbicides (e.g., Aliotta et al. 1996), and antimicrobials (e.g., Mandalari et al. 2007), while others are medically useful (e.g., Holmstedt et al. 1979; Moraes et al. 2003).

Given its great morphological diversity that include a variety of habits, flowers, and fruits, allied with a broad geographic distribution, Rutaceae has been traditionally divided into subfamilies, tribes, and subtribes, following the classifications of Engler (1874, 1896 and 1931, see Chase et al. 1999, and especially Groppo et al. 2008, for a detailed discussion of the these groups). Although subfamily Aurantioideae (*Citrus* and allies) has emerged as monophyletic in recent molecular analyses (e.g., Chase et al. 1999, Scott et al. 2000, Groppo et al. 2008, Bayer et al. 2009), all other subfamilies with more than one genus (Flindersioideae, Rutoideae, and Toddalioideae) and almost all tribes are not monophyletic (Groppo et al. 2008), and rearrangements of the subfamilies have been suggested or proposed (Chase et al. 1999, Scott et al. 2000; Groppo et al. 2008; Kubitzki et al. 2011). Groppo et al. (2008) demonstrated that geographic distribution of the genera could be more relevant than traditionally used characters of the fruit to an understanding of diversification within the family.

One subtribe that had not yet been sampled in molecular phylogenetic studies of the family is Pitaviinae, which comprises a single genus and species, *Pitavia punctata* Molina. (photos can be seen at http://www.florachilena.cl/Niv_tax/Angiospermas/Ordenes/Sapindales/Rutaceae/Pitavia%20punctata/Pitavia%20punctata.htm ). This species is restricted to the temperate forests of the Coastal Cordillera of south-central Chile at 35°-38°S. It is the sole species of Rutaceae native to the continental area of that country (another rutaceous species, *Zanthoxylum mayu* Bertero, is restricted to Juan Fernández Island, see Reiche 1896). Both *Pitavia* and its highly fragmented forest habitat are currently threatened by anthropogenic disturbances, such as clearing of forest for cultivation of wheat and unsustainable extraction of firewood (Newton et al. 2009).

*Pitavia* consists of small trees with simple, opposite to whorled leaves and unisexual, 4-merous, diplostemonous flowers of which the four carpels are proximally connate and joined subapically in a common style. The fruit is composed of one to four fleshy drupes, each with a solitary seed (Engler 1931, Kubitzki et al. 2011). The indehiscent fruit of *Pitavia* differs from the dehiscent, capsular or follicular fruits of all other genera in Zanthoxyleae—the tribe to which *Pitavia* belongs according to Engler (1931), who placed *Pitavia* in a subtribe of its own mainly because of these fruits and its isolated geographic distribution. Although recent molecular studies (e.g., Chase et al. 1999, Poon et al. 2007, Groppo et al. 2008) have shown that the tribe Zanthoxyleae is not monophyletic, *Pitavia* remained unsampled, and Kubitzki et al. (2011) put this genus in a *insertae sedis* position in an infrafamilial classification.

The main objective of this study is to determine the position of Chilean *Pitavia* within a the Rutaceae phylogeny and to assess whether the genus is more closely related to Australasian members of Rutaceae or to Neotropical ones (such as the tribes Galipeeae or Toddalioideae, in Engler's [1931] classification). To examine this, we chose two non-coding regions from cpDNA, the *rps16* gene intronand the *trnL-trnF* region, for a representative sampling of Rutaceae. The type II *rps16* intron was first used for phylogenetic analysis by Oxelman et al. (1997). The *trnL-trnF* region is composed of the *trn*Lintron and the *trnL-trnF* intergenic spacer (Taberlet et al. 1991). Non-coding regions have higher rates of evolution than coding regions; for example, *trnL-trnF* evolved 1.93–11.72 times faster than *rbcL* in certain genera of Gramineae (Gielly and Tabberlet 1994 and references herein). Thus, fragments such as the *rps16* intron and the *trnL-trnF* region have been employed mostly at infrafamilial levels, with good resolution in groups of angiosperms, and have been commonly used in phylogenetic studies of Rutaceae (e.g., Scott et al. 2000, Samuel et al. 2001, Morton et al. 2003, Poon et al. 2007, Groppo et al. 2008, Salvo et al. 2008).

The present study includes genera from all Englerian subfamilies, tribes, and almost all subtribes of Rutaceae and accounts for a broad geographic representation of the family. The large sample of genera used here provides a basis for revising Engler’s circumscription of the subfamilies of Rutaceae. Although new arrangements of subfamilies have been proposed (e.g., Kubitzki et al. 2011, Appelhans et al. 2011), a classification that comprises only monophyletic subfamilies is still needed. This new proposal will serve as a framework for other studies of the family, with the goal of recognizing only monophyletic subfamilies of Rutaceae.

## Methods

Given the uncertain position of *Pitavia* within the Rutaceae phylogeny, representatives of all subfamilies and tribes and almost all subtribes of Rutaceae (sensu Engler 1931, and Swingle and Reece 1967 for tribes and subtribes of Aurantioideae) were sampled (see Appendix 2 and Groppo et al. 2008 for details on Englerian infrafamilial classifications). This sampling is present in the combined matrix used by Groppo et al. (2008) to infer the phylogeny of infra-familial groups in Rutaceae, which comprises almost one third of the estimated number of genera in the family. Sequences from *Cneorum* and *Ptaeroxylon* are also included in the matrix, since these families have been included in Rutaceae (Chase et al. 1999; Groppo et al. 2008, APG III 2009, Appelhans et al. 2010). *Carapa*, *Cedrela*, and *Guarea* (Meliaceae), *Simaba* (Simaroubaceae sensu stricto, Fernando and Quinn 1995), and *Cupania* and *Allophylus* (Sapindaceae), all from families consistently included in Sapindales (Gadek et al. 1996, APG III 2009) were used as outgroups in all analyses. Thus, a total of 61 terminals (including *Pitavia*) were used (55 of Rutaceae in 53 genera, and six of Meliaceae, Sapindaceae, and Simaroubaceae). All DNA sequences are deposited in GenBank, and the accession numbers of the sequences are given in Appendix 2.

Total genomic DNA was extracted from 5 mg of dried leaf sample from a collection of *Pitavia punctata* (*Kubitzki 01-07*, Herbarium HBG) using a modified CTAB protocol (Doyle and Doyle 1987). The *rps16* intron was amplified using *rps*F> and *rps*R2<primers described in Oxelman et al. (1997). The PCR reaction volume (50 mL) contained 30.75μL of water, 3μL of 1% polyvinyl pyrrolidone (PVP), 3μL of MgCl_2_ 50mM (Invitrogen), 5μL of *Taq*Buffer (10x, Fermentas), 5μL of dNTP 10mM (Fermentas), 0.25μL of *Taq*Polymerase (Thermo Scientific), 0.25μL of each primer (Sigma-Aldrich), and 2μL (150–200ng) of DNA sample. Thermal cycling was performed in a PTC-100 Thermal Sequencer (MJ Research Inc., Waltham, Massachusetts, USA), using initial denaturation at 95°C (2 min), followed by 33 cycles at 95°C (30s), 57°C (1min), 72°C (2min), ending with an elongation at 72°C (7min). The *trnL-trnF* region was amplified using trn-c> and trn-f< primers described in Tabberlet et al.(1991). PCR reaction volume (50 μL) contained the same proportions and substances as those used to amplify the *rps16* intron. Thermal cycling was performed using initial denaturation at 94°C (7 min), followed by 30 cycles at 94°C (1min), 56°C (1min), 72°C (1min), ending with an elongation at 72°C (7min). PCR products were purified with GFX^TM^ PCR columns (Amersham Biociences, Piscataway, New Jersey, USA), following the manufacturer’s recommendations. The sequencing reaction volume was 10μL, containing 3.25μL of water, 2μL of BigDye Terminator Ready Reaction (Invitrogen), 0.5μL (10mM) of primers, and 4.25μL of PCR product (60–150ng of DNA). The reactions were performed in an ABI-3100 automatic sequencer (Applied Biosystems-HITACHI), using 25 cycles at 96°C (10s), 50°C (15s), and 60°C (4min). Sequences were assembled and edited using the Biological Sequence Aligment Editor software (BioEdit), v.5.0.9 (Hall 1997–2011). Each fragment was carefully examined to verify concordance among the different reads. Limits of the *trnL-trnF* region and the *rps16* intron were determined by comparison with sequences deposited at GenBank. Sequences of *Pitavia* were then added to the combined matrix of Groppo et al. (2008).

Automated alignments of the sequences were made with Clustal X (Thompson et al. 1997) using default parameters. Indels were treated as missing data. As the study of Groppo et al.(2008) showed a better resolution of the clades in Rutaceae when the *rps16* and *trnL-trnF* results were concatenated, we followed the same approach here. Parsimony analyses were made using PAUP* v.4.0b10 (Swofford 2002) using heuristic search. All characters were unordered and equally weighted (Fitch parsimony, Fitch 1971). Searches were performed with the tree-bisection-reconnection (TBR) branch-swapping algorithm with “steepest descent” and “multrees” options, with 100 random-taxon addition replicates, and with 10 trees held in each replicate. Bootstrap analyses (Felsenstein 1985) were performed to compute support for clades, with 1000 pseudoreplicates (10 trees retained in each pseudoreplicate), random addition of sequences, and TBR branch-swapping.

Bayesian phylogenetic inference was performed with MrBayes v. 3.1.2 (Huelsenbeck and Ronquist 2001, Huelsenbeck et al. 2001, Ronquist and Huelsenbeck 2003) at the Computational Biology Service Unit hosted by Cornell University, USA (http://cbsuapps.tc.cornell.edu ). MrModelTest v. 2.3 (Posada and Crandall 1998, 2001, Nylander 2004) was used to choose the best evolutionary model for the *rps16* and *trnL-trnF* concatenated sequences, as selected by the Akaike Information Criterion. Four independent analyses were run, each performing 10 million generations, sampling every 1000^th^ generation and using 3 heated and 1 cold chain, with temperature 0.2 and other default settings. Tracer v 1.4.1 (Rambaut and Drummond 2007) was used to assess convergence of the runs and to discard the initial 20% of the trees as a burn-in. The remaining 30,000 trees were used to compute a 50% majority-rule consensus phylogram.

## Results

The Akaike Information Criterion implemented in MrModelTest chose the GTR + G + I evolutionary model as the best fit for the *rps16* and *trnL-trnF* concatenated sequences. The burn-in value was set to 4,000 tree samplings, reflecting 2 million generations, i.e., long after the analysis was considered to have stabilized (by inspection of effective sample sizes and standard deviation of split frequencies). The aligned matrix comprised a total of 2,229 characters: 1123 invariable, 467 variable but parsimony-uninformative, and 639 parsimony-informative. At the point when the search was interrupted, parsimony analysis resulted in 10,000 most parsimonious trees with 2,535 steps, consistency index (CI) = 0.68 (0.51 excluding uninformative characters), and retention index (RI) = 0.68.

**Figure 1.**
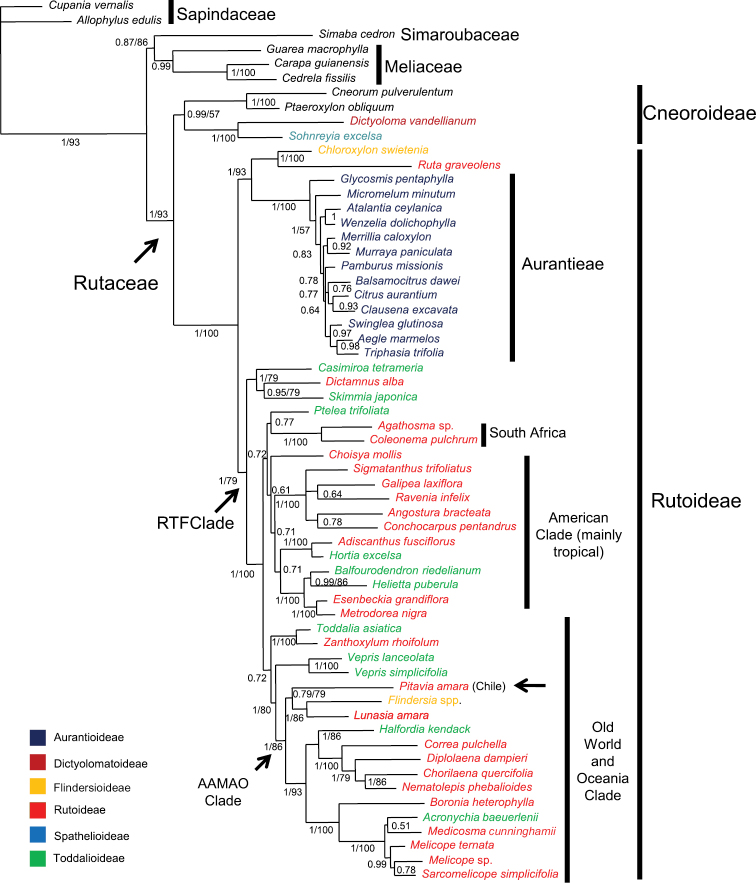
Majority-rule consensus tree of Rutaceae estimated using Bayesian inference on a combined *rps16* and *trnL-trnF* dataset. Posterior probabilities (PP ≥50%) are shown above branches. Bootstrap percentages (BP, only for branches in agreement with those obtained in the Bayesian analysis) follow posterior probabilities; when only one number appears supporting a clade it refers to Bayesian posterior probabilities. Taxon names are color-coded to indicate their Englerian assignment to subfamilies. A new proposal that recognizes monophyletic groups (subfamilies Cneoroideae and Rutoideae and tribe Aurantieae) is indicated by the vertical bars. The position of *Pitavia punctata*, as well as the Rutaceae, the “RTF” (from Rutoideae, Toddalioideae and *Flindersia*) and “AAMAO” (”African-Asian-Malesian-Astralasian-Oceanic”) clades (see text) are indicated by arrows. Note: *Zanthoxylum* is pantropical.

The majority-rule consensus tree with posterior probabilities (PP) that was estimated using Bayesian Inference is shown in Fig. 1. Bootstrap percentages (BP) are also shown for clades recovered in the majority-rule consensus tree of the bootstrap analysis. As commonly seen in the literature, a higher resolution was obtained with the Bayesian analysis than with the majority-rule bootstrap consensus trees based on parsimony, as can be noted in the figure: many clades that appeared in the 50% majority-rule Bayesian tree do were not recovered in the boostrap analysis. (even in clades with PP as high as 0.99). Given its better resolution and branch support values, and the generally accepted superiority of Bayesian methods in inferring reliable phylogenetic relationships we chose to discuss our results on the basis of the Bayesian tree.

Topology of the Bayesian analysis was congruent with that obtained in the study of Groppo et al.(2008) using parsimony, but with a better resolution of some clades as discussed above. Rutaceae appeared as monophyletic (PP=1, BP=93), encompassing two internal clades, one with *Cneorum*, *Ptaeroxylon*, *Sohnreyia*, and *Dictyoloma* with mixed support (strong PP=0.99 and weak BP=57) and another with the remaining Rutaceae (1/100). This clade is divided in two sister-groups: one (1/93) formed by *Chloroxylon* (Flindersioideae) plus *Ruta* (Rutoideae) and all Aurantioideae (the only monophyletic Englerian subfamily with more than one genus) and the other (1/79) with interdigitated representatives of Rutoideae (without Ruteae), Toddalioideae, and *Flindersia* (Flindersioideae), the RTF Clade. Chilean *Pitavia* appears as part of this last group, in a clade containing also *Lunasia* and *Flindersia* (1/86), which in turn is part of a larger clade (1/86) formed by representatives of Rutaceae of Old World, Australasia and Oceanic islands from Pacific (the ”African-Asian-Malesian-Astralasian-Oceanic - AAMAO clade”)

## Discussion

### Phylogenetic and biogeographic relationships of *Pitavia*

A clade comprising *Flindersia* and *Lunasia* was obtained also by Chase et al. (1999), using *rbcL* and *atpB* sequence data and by Groppo et al.(2008) *rps16* and *trnL-trnF*. The present results indicate that *Pitavia* is sister to this clade. *Flindersia* (17 species, in Australia, Moluccas, to Irian and Papua) and *Lunasia* (one species, *Lunasia amara* Blanco, in NE Australia, New Guinea, Philippines, and the Malayan region) differ in several morphological characteristics, discussed in detail by Groppo et al.(2008). Because of its capsular fruits with winged seeds and its compound leaves, *Flindersia* was positioned by Engler (1931) in subfamily Flindersioideae, together with the genus *Chloroxylon*, whose winged seeds have to be interpreted as a convergence given the position of these two genera in the phylogeny. *Lunasia*, however, has simple leaves and features common within the Englerian subfamily Rutoideae, such as follicular fruits with elastic endocarp and unwinged seeds. Additionally, the trimerous flowers in *Lunasia*, these disposed in congested glomerules, are a unique combination of characteristics within Rutaceae. Comparing the capsular fruits and winged seeds of *Flindersia* with the follicular fruits and unwinged seeds of *Lunasia* and the indehiscent fleshy drupelets of *Pitavia* shows that indehiscent fruits and winged seeds have appeared more than once within the evolutionary history of the family (Groppo et al. 2008). In fact, in Rutaceae groups of genera with indehiscent fruits occur frequently as sisters to others with dehiscent ones (*e.g., Acronychia* and *Melicope* or *Adiscanthus* and *Hortia*, see Groppo et al. 2008). Studies of fruit development in the family have demonstrated differences in formation of dehiscent (Hartl 1957) and indehiscent fruits (Hartl 1957, Zavaleta-Mancera and Engleman 1991). Groppo et al. (2008) showed that large clades of Rutaceae correspond better with their geographic distributions than with gross fruit morphology.

The morphological resemblance of *Pitavia* to Rutaceae from Oceania and Southern Asia was implicitly suggested by Hartley (1997), when he gave the generic name *Pitaviaster* (monoespecific, from Eastern Australia) to the species *Pitaviaster haplophyllus* (F.Müell.) T.G.Hartley, segregated from *Euodia* (seven species in New Guinea, North-Eastern Australia eastwards to Samoa and Niue), givenits general resemblance to *Pitavia*. *Pitaviaster* and *Pitavia* share opposite to whorled, simple leaves, axillary inflorescences, rather small, 4-merous flowers, and fruit composed of 1-4 fleshy drupelets. They differ, however, especially on fruit characters: *Pitaviaster* with woody mesocarp (vs. fleshy in *Pitavia*) and cartilaginous (vs. thin, ligneous, see Kubitzki et al. 2011, p.: 342) endocarp. Even though aware of these similarities, Hartley (1997) hypothesized that *Pitavia* was nearest to *Acronychia*,given other morphological characteristics of flower and fruit. As shown in the present study, the close relationship of *Acronychia* to other genera as *Medicosma*, *Sarcomelicope*, and *Melicope*, all of them (including *Acronychia*) from Australasia, Southern Asia, Oceanic or even African/Malagasy or Indo-Himalayan (as some species of *Melicope*) regions, but relatively distant from *Pitavia*,does not corroborate a close relationship between *Acronychia* and *Pitavia*.

The association of Chilean *Pitavia* with *Flindersia* and *Lunasia* is an example of biogeographical affinity between components of the faunas and floras occurring on both sides of the Pacific Ocean especially in the Southern Hemisphere (for a reviews of this issue and examples, see Grehan 2007 and Heads 2012). *Pitavia* is restricted to temperate forests of Chile where other taxa that have Trans-Pacific distributions occur, such as species of *Nothofagus* (Fagaceae), found in Chile and eastern Argentina as well as in New Caledonia, New Guinea, Australia, Tasmania, and New Zealand (Humphries 1981), and some members of the Proteaceae (Barker et al. 2007).

Distributional patterns in Rutaceae have often been explained on basis of vicariance events (e.g., Hartley 2001a, 2001b; Ladiges and Cantrill 2007), and age estimates for disjunctions have been calibrated against the timing of plate movements suggested by geologists (Kubitzki et al. 2011). The disjunct distributions in the Southern Hemisphere have been explained in terms of vicariant events linked with the break-up of the supercontinent Gondwana over the past 160 million years (Sanmartín and Ronquist 2004). Indeed, Heads (2012, on p. 427), based mostly on the phylogeny presented by Groppo et al. (2008), presents the hypothesis in which “the high diversity of groups such as Rutaceae in Brazil, South Africa, Western Australia, New Caledonia, and the Hawaiian Islands is the direct result of phylogeny and vicariance producing allopatric, regional blocks of taxa.” In this hypothesis of vicariance, events linked to the separation of land masses in the South Hemisphere would explain the link between Chilean *Pitavia* and other genera from Pacific Islands, Australasia, and portions of Asia. This view can be reinforced by the fact that fossils of Rutaceae (seeds, fruits, wood, and leaves, Gregor 1989), classified as form genera *Rutaspermum*, *Toddaliospermum*, and *Pteleaecarpum*,are dated from the Cretaceous to Palaeocene (100 mybp as the age of first ’doubtful’ Rutaceae fossils and 80 mybp as first *Rutaspermum* fossils). Various species accommodated in *Rutaspermum* may represent *Zanthoxylum* (Tiffney 1980), and others may represent *Tetradium* (Hartley 2001a, 2001b). As noted by Kubitzki et al. (2011), the oldest fossils of the family (*Tetradium*, *Toddalia*, *Zanthoxylum*) belong to the group of five genera that produce 1-btiq alkaloids and that are specialized for bird dispersal. The appearance of these genera in the Palaeocene sets a minimum age of Rutaceae, and the family may have originated earlier. Therefore, the hypothesis that a longer period of time was required to isolate *Pitavia* from related groups in the Pacific, Australasia, and Asia could appear reasonable.

Estimates of the age of Rutaceae based on molecular studies vary from 37 to 93.3 mybp (Muellner et al. 2003, Pfeil and Crisp 2008, Wilkström et al. 2001, Appelhans et al. 2012a), but many times these assumptions of age based on molecular dating conflict with a vicariance explanation for the observed distribution patterns in the family. Several authors have therefore explained some disjunct distributions in the family on terms of long-distance dispersal, e.g., the Aurantioideae's colonization of New Caledonia from other land masses (Pfeil and Crisp 2008). SanMartín and Ronquist (2004), discussing South Hemisphere distributions of groups of plants and animals, stated that there has been land connections between South America and Australia via Antarctica until about 35mybp. The same authors mention that the biogeographical patterns in the plant lineages they studied have not been significantly influenced by Gondwanan breakup. If this idea is correct, disjunction between *Pitavia* and its relatives in the Pacific Islands, Oceania, and Old World (e.g., Southern Asia), could be more recent than the Gondwana break-up, with Island conections allowing long-distance dispersion. Fleshy fruits of *Pitavia* suggest a dispersal by animals, and birds (or even bats) could act as dispersers in the past. Further molecular dating studies, in combination with further evaluation and integration of the fossil record, could help clarify the conflicting suggestions of vicariance or long-distance dispersal to explain the disjunction of *Pitavia* from its sister groups.

Despite the linking of *Flindersia*, *Lunasia*, and *Pitavia* shown in the present study, it is premature to say that *Pitavia* is indeed sister to the *Flindersia*/*Lunasia* clade because many genera, such as *Dinosperma*, *Perryodendron*, *Pitaviaster*, *Crossosperma*, and *Dutailliopsis*, have not yet been included in phylogenetic studies. However, the support (PP PP, 86% of BP) of the clade (*Pitavia* (*Flindersia*, *Lunasia*)) is strong enough to refute an association of *Pitavia* with the clade with *Acronychia*, *Melicope*, and *Sarcomelicope* (as suggested by Hartley 1997, see above).

### Chloroxylon

The placement of *Chloroxylon* (Flindersioideae) near *Ruta* is supported by the possession of diplostemonous flowers, unguiculate petals with concavities embracing the smaller antepetalous stamens, a developed urceolate disc, and more than two ovules per locule. The base chromosome number in both genera is X=10 (Stace et al. 1993), a number so far encountered elsewhere in Rutaceae only in *Boenninghausenia*, in the same subtribe (Rutinae) as *Ruta*. Thus, the base number X=10 may be a synapomorphy of this clade. *Ruta* and its allies in Rutinae, however, are characterized by an herbaceous or suffrutescent habit and unwinged seeds, and *Chloroxylon* by arborescent habit and winged seeds. The relationship of *Ruta* and *Chloroxylon*, indicated in the present study and in Morton et al. (2003), Chase et al. (1999), and Groppo et al. (2008) is based on sequences obtained from samples of the same collection (*Chase 1291*, K). Samples from additional collections of *Chloroxylon* are needed to further test its relationship with *Ruta*.

### A new classification of subfamilies in Rutaceae

Another objective of this work is to present a new proposal to replace the standard classification of subfamilies of Rutaceae, replacing that proposed by Engler (1874, 1896, 1931). In his last system of classification, Engler (1931) recognized seven subfamilies that were further divided into tribes and subtribes. As discussed by Groppo et al.(2008), Englerian subfamilies were defined mainly by degree of connation and number of carpels, fruit structure, and gland histology. Although the monogeneric subfamily Rhabdodendroideae was excluded from Rutaceae (Prance 1968, 1972, Fay et al. 1997), the remaining subfamilies, Aurantioideae, Dictyolomatoideae, Flindersioideae, Rutoideae, Spathelioideae, and Toddalioideae continued to be recognized based on characteristics of the subfamilies which were discussed in detail in Chase et al. (1999) and Groppo et al. (2008). Several studies of morphology (e.g., Hartley 1974, 1981, 1982), chromosome number (Stace et al. 1993), secondary metabolites (Da Silva et al. 1988), and more recently, molecular data (Chase et al. 1999, Poon et al. 2007, Groppo et al. 2008) have demonstrated the need for a better circumscription of the subfamilies. To further evaluate Engler’s circumscriptions of the groups, the broadest sampling of Rutaceae to date, i.e., 53 genera representing all subfamilies, tribes, and almost all subtribes, was included in the study by Groppo et al. (2008) and the present one. Although Aurantioideae has emerged as a monophyletic group, all other subfamilies with more than one genus appeared as not monophyletic (see Fig. 1). The genera of Toddalioideae and Rutoideae appeared in mixed clades here and in previous studies (Chase et al. 1999, Scott et al. 2000, Poon et al. 2007, Groppo et al. 2008) and are clearly not monophyletic. The position of *Ruta* (and remaining Ruteae), far from other Rutoideae and near the Aurantioideae, has been obtained in all studies that included genera of Aurantioideae.

Given these data, realignments of the infrafamilial groups in Rutaceae have been recently made. In a survey of secondary metabolites (largely influenced by the conclusions of Waterman and Grundon 1983), Da Silva et al. (1988) proposed the rejection of Toddalioideae and its inclusion in Rutoideae (as later did Quader et al. 1991 and Thorne 1992) and several “informal tribes.” Chase et al. (1999), Scott et al. (2000), and Groppo et al. (2008) suggested different circumscriptions of monophyletic subfamilies. One of the concordances among these three studies is the recognition of the *Spathelia*/*Ptaeroxylon* clade as a subfamily, named Spathelioideae (Appelhans et al. 2011) or Cneoroideae (Thorne and Reveal 2007, Kubitzki et al. 2011). Based on priority, Cneoroideae is the correct name for this group (see Appendix 1), which encompasses the Englerian subfamilies Spathelioideae and Dictyolomatoideae and the families Ptaeroxylaceae and Cneoraceae, all recognized as Rutaceae in APG III (2009). A new tribal classification of Cneoroideae was presented by Appelhans et al. (2011, as Spathelioideae).

The remaining Rutaceae or “core Rutaceae” (Groppo et al. 2008, Kubitzki et al. 2011) are here lumped into a single subfamily, Rutoideae, sister to Cneoroideae. In this new circumscription, Rutoideae encompasses Englerian Rutoideae, Toddalioideae, Flindersioideae, and Aurantioideae–a total of 148 genera and approximately 2061 species (Table 1). Thorne (1992), Quader et al. (1991), and Appelhans et al. (2011) previously proposed the inclusion of Toddalioideae in an expanded Rutoideae, but none formally proposed the inclusion of Aurantioideae. Although continued recognition of Aurantioideae as a subfamily might be convenient to the economically important *Citrus* industry, phylogenetic studies [the present one as well as those of Chase et al. (1999), Scott et al. (2000), Groppo et al. (2008), and Appelhans et al. (2011)] show that *Ruta* is much closer to Aurantioideae than to other Rutoideae.

**Table 1.** A summary of the new circumscription of subfamilies in Rutaceae proposed in this study. Approximate number of genera and species and distribution taken from Kubitzki et al.(2011) and Appelhans et al. (2011, for Cneoroideae). RTF = clade comprising Rutoideae, Toddalioideae, and *Flindersia*.

**Subfamily**	**Circumscription**	**Approximate number of genera/ (species)**	**Distribution**
Cneoroideae	Englerian Dictyolomatoideae and Spathelioideae (Engler 1931), plus Cneoraceae, Ptaeroxylaceae (see Appelhans et al. 2011 and 2012a for further details)	8 (35)	Pantropical, extending to subtropical regions in Europe
Rutoideae	Englerian Aurantioideae, Flindersioideae, Rutoideae, and Toddalioideae (Engler 1931)	114 (1770) in clade RTF; 26 (206) in Aurantieae; 7 (84) in Ruteae (including *Cneoridium* and *Haplophyllum*), plus 1 (1) in *Chloroxylon*. Total: 148 (2061)	Pan-tropical, some in temperate or desert areas worldwide
Total		156 (2096)	

**Figure 2.**
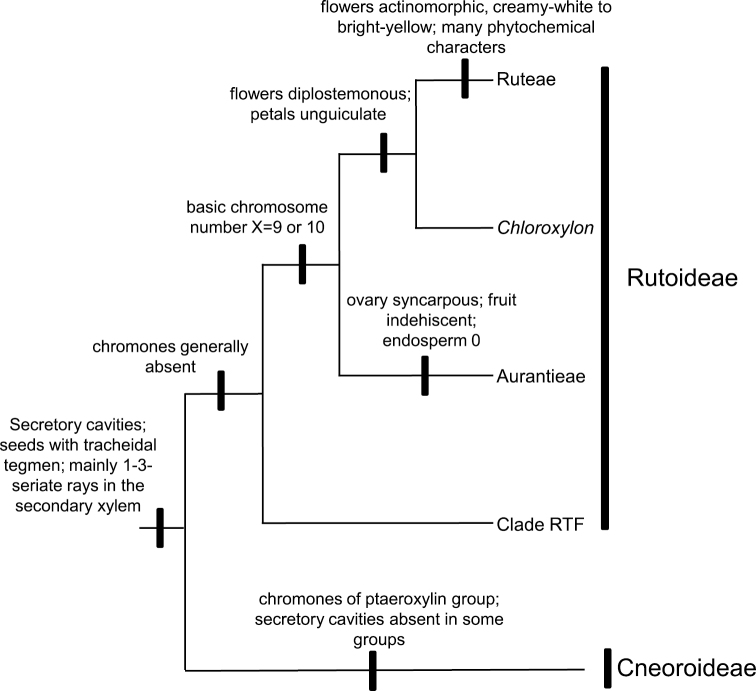
A summary of the phylogenetic relationships of proposed subfamilies in Rutaceae, with some non-molecular characteristics plotted onto a simplified cladogram based on the Bayesian tree. *Chloroxylon* is doubtfully attached to Aurantieae. *Haplophyllum* and *Cneoridium* (both from Englerian Ruteae but closer to Aurantieae that to remaining Ruteae, see Salvo et al. 2010) are missing. *Amyris* (from Englerian Toddalioideae), also close to Aurantieae (unpublished results) is also missing. The RTF clade corresponds to the bulk of Englerian Rutoideae, plus Toddalioideae and *Flindersia*. For discussion of characteristics see Waterman and Grundon (1983), Stace et al. (1993), Groppo et al. (2008), and Appelhans et al. (2011, 2012b).

Restricting the name Rutoideae to *Ruta* and its allies in tribe Ruteae (excluding *Dictamnus*, see Salvo et al. 2008) to preserve Aurantioideae is one option. However, as *Cneoridium* and *Haplophyllum* (not sampled here), both Ruteae, appear to be closer to Aurantioideae than to *Ruta* (see Salvo et al. 2010), it will be necessary also to erect subfamilial names to these two groups. Besides, preservation of Aurantioideae and a narrow Rutoideae would require a different subfamilial name for one of the major clades in Rutaceae, here called “clade RTF“ (Figs 1 and 2), that comprises the bulk of Englerian Rutoideae, Toddalioideae, and *Flindersia* (from Englerian Flindersioideae), a total of 114 genera and 1770 species (Table 1). Appelhans et al.(2011) used the name Toddalioideae (from 1869) for this large clade, unaware that Diosmideae (based on *Diosma*) and Zanthoxyloideae (both from 1832), have priority over Toddalioideae. Yet, the correct choice of a formal name for clade RTF is further complicated by as yet unpublished results of molecular studies by the first author, in which *Amyris*, placed by Engler (1931) in Toddalioideae, appears to be more closely related to Aurantioideae. Were *Amyris* and the Aurantioideae to be recognized as a subfamily, its correct name would be Amyridoideae (published in 1824) rather than Aurantioideae (from 1836). Alternatively, it can be treated within the Rutoideae as the monophyletic tribe Aurantieae, the name proposed by Bentham and Hooker (1862) and the group recognized by Engler (1896, 1931) as the only tribe in the Aurantioideae.

Formal recognition of the expanded Rutoideae and the Cneoroideae at the family level (i.e., respectively as Rutaceae *sensu stricto* and Cneoraceae) is at odds with shared morphological characters. One synapomorphy uniting these two clades, despite its absence in some Cneoroideae (due to a secondary loss, Appelhans et al. 2011), is the presence of secretory cavities containing aromatic ethereal oils in almost all organs (Groppo et al. 2008, Groppo 2010), a feature unique to Rutaceae within the Sapindales. Another putative synapomorphy encountered commonly in the expanded Rutoideae (see Corner 1976 and Johri et al. 1992) and in Cneoroideae (though absent in some, again due to a secondary loss, Appelhans et al. 2011) is the presence of a tracheidal tegmen in the seeds. Additionally, Appelhans et al. (2012b) discussed some wood anatomical characters shared by Spathelioideae (here Cneoroideae) and remaining Rutaceae, as the mainly 1-3-seriate rays in the secondary xylem. Figure 1 presents our chosen classification of the Rutaceae superimposed on that of Engler (1931), and Figure 2 summarizes some characteristics and putative synapomorphies of the major internal groups in the family. A summary of the circumscriptions of the two subfamilies recognized in this study is given at the end of the text.

The classification scheme presented here, with only two monophyletic subfamilies, Cneoroideae and Rutoideae, is a framework for further studies of the family. The next step is the re-circumscription of groups below the subfamilial level, i.e., the tribes and subtribes, as Appelhans et al. (2011) has done for Spathelioideae (here Cneoroideae) and Salvo et al.(2008) for Rutinae. Another challenge is to search for morphological synapomorphies of groups within Rutoideae (especially the “RTF clade”) and to include additional sampling in phylogenetic studies, such as other sequences from monospecific *Chloroxylon*, which appeared, somewhat doubtfully at this point, close to *Ruta* and to Aurantioideae in some studies. Ongoing studies of Neotropical Rutaceae, especially Galipeeae (Rutoideae), conducted by the authors are also expected to change our view of the traditional groups in the family and contribute to the understanding of the phylogeny of the large and widespread Rutaceae.

## Appendix 1

Summary of the subfamilies recognized in the present circumscription of Rutaceae. Source of dates of publication is Reveal, J. “Suprafamilial Names of Extant Vascular Plants,” available at http://www.plantsystematics.org/reveal/pbio/fam/hightaxa7.html

(for a complete list of synonyms see Thorne and Reveal 2007).

**Rutaceae** A.Juss., Gen Pl.: 296. 4 Ago 1789. (type: *Ruta* L.) *nomen conservandum*

**Subfamily Cneoroideae**  Webb, London J. Bot. 1: 257. 1 Mai 1842. (Cneoreae). (type: *Cneorum* L.) [Circumscription: Englerian Dictyolomatoideae and Spathelioideae (Engler 1931) and Cneoraceae and Pteroxylaceae (see Appelhans et al. 2011 for a list of genera and tribal delimitation)]

**Synomyms:**

*Cneoraceae* Vest, Anleit. Stud. Bot.: 267. 1818. (type: *Cneorum* L.)

*Dictyolomatoideae* (“Dictyolomoideae”) Engl. in Engl. and Prantl, Nat. Pflanzenfam. III, 4: 111. Mar 1896. (type: *Dictyoloma* A. Juss.).

*Ptaeroxylaceae* J.-F.Leroy, J. Agric. Trop. Bot. Appl. 7: 456. 1960. (type: *Ptaeroxylon* Radlk.).

*Spathelioideae* Engl. in Engl. and Prantl, Nat. Pflanzenfam. III, 4: 111. Mar 1896. (type: *Spathelia* L.).

**Subfamily Rutoideae** Arn., Encycl. Brit., ed. 7, 5: 104. 9 Mar 1832 (Ruteae). (type: *Ruta* L.) [Circumscription: Englerian Aurantioideae, Flindersioideae, Rutoideae, and Toddalioideae, see Engler (1931)]

**Synomyms:**

Aurantioideae Eaton, Bot. Dict., ed. 4: 39. Apr-Mai 1836 (Aurantiaceae). [(type: *Aurantium* L. (=*Citrus* L.)]. Recognized here as tribe Aurantieae Rchb., Fl. Germ, Excurs. 292). 840. 1832.

(note: see Mabberley (1998) for reasons why Aurantioideae instead of the earlier name Citroideae is to be used).

*Flindersiaceae* C.T.White ex Airy Shaw, Kew Bull. 18: 257. 8 Dec 1964. (type: *Flindersia* R. Br.).

*Flindersioideae* Luerss., Handb. Syst. Bot. 2: 681. Jun 1881 (“Flindersieae”). (type: *Flindersia* R. Br.).

*Toddalioideae* K.Koch, Dendrologie 1: 564. 1869 (“Toddalieae”). (type: *Toddalia* A.Juss.).

## Appendix 2

Voucher information and Genbank accession numbers for taxa used in this study. Missing sequences are marked by “—.“ Arrangement of terminals in Rutaceae follows the new subfamilial delimitation proposed here. For classification of terminals in Engler (1931) and Swingle and Reece (1967, for “Aurantioideae”) see Groppo et al. (2008).

**Genbank accessions:** *trnL-trnF, rps16*.

**Rutaceae**

**Subfamily Cneoroideae**

*Cneorum pulverulentum* Vent. — EU853787, EU853733. *Dictyoloma vandellianum* A. Juss. — EU853793, EU853739. *Ptaeroxylon obliquum* (Thunb.) Radlk. — EU853812, EU853762. *Sohnreyia excelsa* Krause — EU853820, EU853770.

**Subfamily Rutoideae**

*Acronychia baeuerlenii* T. G. Hartley — EU853774, EU853719. *Adiscanthus fusciflorus* Ducke — EU853775, EU853721. *Aegle marmelos* (L.) Corrêa ex Roxb. — AY295294, AY295268. *Afraegle paniculata* (Schum. and Thonn.) Engl. — AY295295, AY295269. *Agathosma* sp. — EU853776, EU853722. *Angostura bracteata* (Engl.) Kallunki — EU853778, EU853724. *Atalantia ceylanica* (Arn.) Oliv. — AY295288, AY295262. *Balfourodendron riedelianum* (Engl.) Engl. — EU853779, EU853725. *Balsamocitrus dawei* Stapf — AY295278, AY295252. *Boronia heterophylla* F. Muell. — EU853780, EU853726. *Casimiroa tetrameria* Millsp. — EU853782, EU853728. *Chloroxylon swietenia* DC. — AY295276, AY295250. *Choisya mollis* Standl. — EU853784, EU853730. *Chorilaena quercifolia* Endl. — EU853785, EU853731. *Citrus aurantium* L. — EU853786, EU853732. *Clausena excavata* Burm. f. — AY295284, AY295258. *Coleonema pulchrum* Hook. — EU853788, EU853734. *Conchocarpus pentandrus* (A.St.-Hil.) Kallunki and Pirani — EU853735, EU853735. *Correa pulchella* Mackay ex Sweet — EU853790, EU853736. *Dictamnus albus* L. — EU853792, EU853738. *Diplolaena dampieri* Desf. — EU853794, EU853740. *Esenbeckia grandiflora* Mart. — EU853795, EU853741. *Flindersia australis* R. Br. — AF038628 (intron) AF026009 (spacer), —,— spacer. *Flindersia collina* F. M. Bailey — —, EU853742. *Galipea laxiflora* Engl. — EU853796, EU853743. *Halfordia kendack* (Montrouz.) Guillaumin — EU853798, EU853745. *Hortia excelsa* Ducke — EU853801, EU853748. *Glycosmis pentaphylla* (Retz.) Corrêa — AY295279, AY295253. *Helietta puberula* R. E. Fries — EU853799, EU853746. *Lunasia amara* Blanco — EU853805, EU853753. *Medicosma cunninghamii* (Hook.) Hook. f. — EU853806, EU853754. *Melicope ternata* J. R. Forst. and G. Forst. — EU853808, EU853756. *Melicope* sp. — EU853807, EU853755. *Metrodorea nigra* A. St.-Hil. — EU853809, EU853757. *Micromelum minutum* (G. Forst.) Wight and Arn. — AF025520, AF320266. *Murraya paniculata* (L.) Jack — EU853810, EU853758. *Nematolepis phebalioides* Turcz. — AF025522 (spacer), EU853759. *Pitavia amara* Blanco —KC261635, KC261636. *Ravenia infelix* Vell. — EU853814, EU853764. *Ruta graveolens* L. — EU853815, EU853765. *Pamburus missionis* (Wight) Swingle — AY295300, AY295274. *Ptelea trifoliata* L. — EU853813, EU853763. *Sarcomelicope simplicifolia* (Endl.) T. G. Hartley — EU853816, EU853766. *Sigmatanthus trifoliatus* Huber ex Emmerich — EU853817, EU853767. *Skimmia japonica* Thunb. — EU853819, EU853769. *Swinglea glutinosa* (Blanco) Merr. — AY295285, AY295259. *Toddalia asiatica* (L.) Lam. — EU853821, AF320278. *Triphasia trifolia* (Burm. f.) P. Wilson — EU853822, AY295271. *Vepris lanceolata* (Lam.) G. Don — EU853823, EU853771. *Vepris simplicifolia* (Engl.) W. Mziray — EU853824, EU853772. *Wenzelia dolichophylla* (Lauterb. and K. Schum.) Tanaka — AY295286, AY295260. *Zanthoxylum rhoifolium* Lam. — EU853773, EU853720.

**Meliaceae**

*Carapa guianensis* Aubl. — EU853781, EU853727. *Cedrela fissilis* Vell. — EU853783, EU853729. *Guarea macrophylla* Vahl — EU853797, EU853744.

**Sapindaceae**

*Allophylus edulis* (A. St.-Hil.) Niederl. — EU853777, EU85853723. *Cupania vernalis* Cambess. — EU853791, EU853737.

**Simaroubaceae**

*Simaba cedron* Planch. — EU853818, EU853768.
